# Region-specific mechanisms of corticosteroid-mediated inotropy in rat cardiomyocytes

**DOI:** 10.1038/s41598-020-68308-4

**Published:** 2020-07-14

**Authors:** Caroline Wacker, Niklas Dams, Alexander Schauer, Anne Ritzer, Tilmann Volk, Michael Wagner

**Affiliations:** 1 Institut für Zelluläre und Molekulare Physiologie, Friedrich-Alexander-Universität Erlangen-Nürnberg, Waldstraße 6, 91054 Erlangen, Germany; 2 Muscle Research Center Erlangen (MURCE), Friedrich-Alexander-Universität Erlangen-Nürnberg, Erlangen, Germany; 3 Abteilung für Rhythmologie, Herzzentrum Dresden, Fetscherstraße 76, 01307 Dresden, Germany

**Keywords:** Physiology, Cardiovascular biology, Circulation, Ion transport, Cardiovascular biology

## Abstract

Regional differences in ion channel activity in the heart control the sequence of repolarization and may contribute to differences in contraction. Corticosteroids such as aldosterone or corticosterone increase the L-type Ca^2+^ current (I_CaL_) in the heart via the mineralocorticoid receptor (MR). Here, we investigate the differential impact of corticosteroid-mediated increase in I_CaL_ on action potentials (AP), ion currents, intracellular Ca^2+^ handling and contractility in endo- and epicardial myocytes of the rat left ventricle. Dexamethasone led to a similar increase in I_CaL_ in endocardial and epicardial myocytes, while the K^+^ currents I_to_ and I_K_ were unaffected. However, AP duration (APD) and AP-induced Ca^2+^ influx (Q_Ca_) significantly increased exclusively in epicardial myocytes, thus abrogating the normal differences between the groups. Dexamethasone increased Ca^2+^ transients, contractility and SERCA activity in both regions, the latter possibly due to a decrease in total phospholamban (PLB) and an increase PLBpThr17. These results suggest that corticosteroids are powerful modulators of I_CaL_, Ca^2+^ transients and contractility in both endo- and epicardial myocytes, while APD and Q_Ca_ are increased in epicardial myocytes only. This indicates that increased I_CaL_ and SERCA activity rather than Q_Ca_ are the primary drivers of contractility by adrenocorticoids.

## Introduction

Aldosterone, a corticosteroid hormone of the adrenal gland and a key player in the renin–angiotensin–aldosterone cascade, has a pivotal role in maintaining total body Na^+^ via its action on the mineralocorticoid receptor (MR) in epithelia that control excretion of NaCl such as the aldosterone sensitive distal nephron, the colon, or sweat ducts. Aldosterone thereby controls extracellular fluid volume and arterial blood pressure^1^. Beyond its action on Na^+^-absorbing epithelia, the effects of MR activation on various non-epithelial targets, such as the heart, are increasingly recognized. However, its mechanisms of action on these non-classical targets are far less understood. Clinical trials provide convincing evidence that MR antagonistic therapy increases survival in patients with heart failure, by, among others, decreasing the rate of fatal arrhythmias^2,3^, which are facilitated by electrical remodeling of the myocardium^4^. In line with this observation, a number of studies have demonstrated that MR activation modulates ionic currents and transporters in ventricular cardiomyocytes, including the fast Na^+^ current^5^, the hyperpolarization-activated and cyclic-nucleotide gated current^6^, the ryanodine receptor^7^, and the L-type Ca^2+^ current (I_CaL_)^8^. The latter is of particular importance as alterations in I_CaL_ not only affect cardiac electrophysiology and contractility directly, but also serve as a first step in chronic electrical and mechanical remodeling of the heart^9,10^. Consequently, mice overexpressing the MR show electrical abnormalities, arrhythmia and cardiac hypertrophy^11^.

In the normal heart, repolarization is a well-coordinated process controlled by the sequence of excitation and regional differences in action potential duration (APD) among the ventricles. In fact, the regions excited early, such as endocardial or septal parts of the left ventricle, repolarize last due to longer action potentials (AP) and vice versa^12^. Although differences in a number of ionic currents contribute to the heterogeneity in APD among the ventricle^13,14^, differences in the magnitude of the transient outward K^+^ current (I_to_) between endo- and epicardial layers of the ventricle are the most prominent. In rodents such as mice and rats, these differences in I_to_ are mainly responsible for the gradient in APD among the left ventricular free wall^15,16^, while in larger mammals such as dog or humans, I_to_ magnitude exerts less influence on total APD, but rather controls the membrane voltage level during the early part of the plateau phase^17–19^. Since the AP constitutes the driving force for the ionic currents flowing during the AP, differences in AP waveform directly affect ion fluxes across the membrane. This is particularly important for the L-type Ca^2+^ current, as the amount of Ca^2+^ entering the myocytes during an AP controls excitation–contraction–coupling. For example, despite a similar I_CaL_ magnitude in endo- and epicardial myocytes, the longer AP in endocardial myocytes leads to an increased AP-induced Ca^2+^ influx^20^. Differences in duration and shape of the AP, therefore, not only control the sequence of repolarization but also affect Ca^2+^ influx, intracellular Ca^2+^ cycling, contractility and cardiac remodeling^21–23^. The particular relevance of the AP shape is highlighted by studies, in which epicardial myocytes displayed increases in Ca^2+^ influx, Ca^2+^ transients and contractility, when clamped on an endocardial AP and vice versa^24^. Moreover, alterations in AP waveform in ferret myocytes caused desynchronized SR Ca^2+^ release which is typically observed in myocytes originating from failing hearts^25^. Although the magnitude of I_to_ is the major parameter responsible for differences in AP shape and duration among different regions of the ventricular wall, it is important to note that the AP waveform of individual myocytes is a function of the cell’s magnitude of both, I_to_ and I_CaL_. In experiments, in which I_to_, I_CaL_ and APD were measured in the same cells, Gomez and coworkers showed that early repolarization of the rat AP (APD_20_) neither correlated with the magnitude of I_CaL_ nor with I_to_ alone, but only with both currents together^26^. This suggests that changes not only in the magnitude of I_to_, but also in I_CaL_ might substantially affect AP waveform and in turn Ca^2+^ influx, Ca^2+^ transients and contractility.

In the present study, we therefore hypothesized that the corticosteroid induced increase in I_CaL_ affects AP shape, Ca^2+^ influx, Ca^2+^ transient and contractility differently in endo- and epicardial myocytes. To address this question, we investigated the influence of mineralocorticoid receptor activation on I_CaL_, AP-induced Ca^2+^ influx, contractility, Ca^2+^ transient and SR Ca^2+^ handling in endo- and epicardial myocytes isolated from the rat left ventricle. We show that MR activation substantially increases contractility to a similar extent in both endo- and epicardial myocytes but modulates intracellular Ca^2+^ handling differently.

## Results

**Figure 1:**
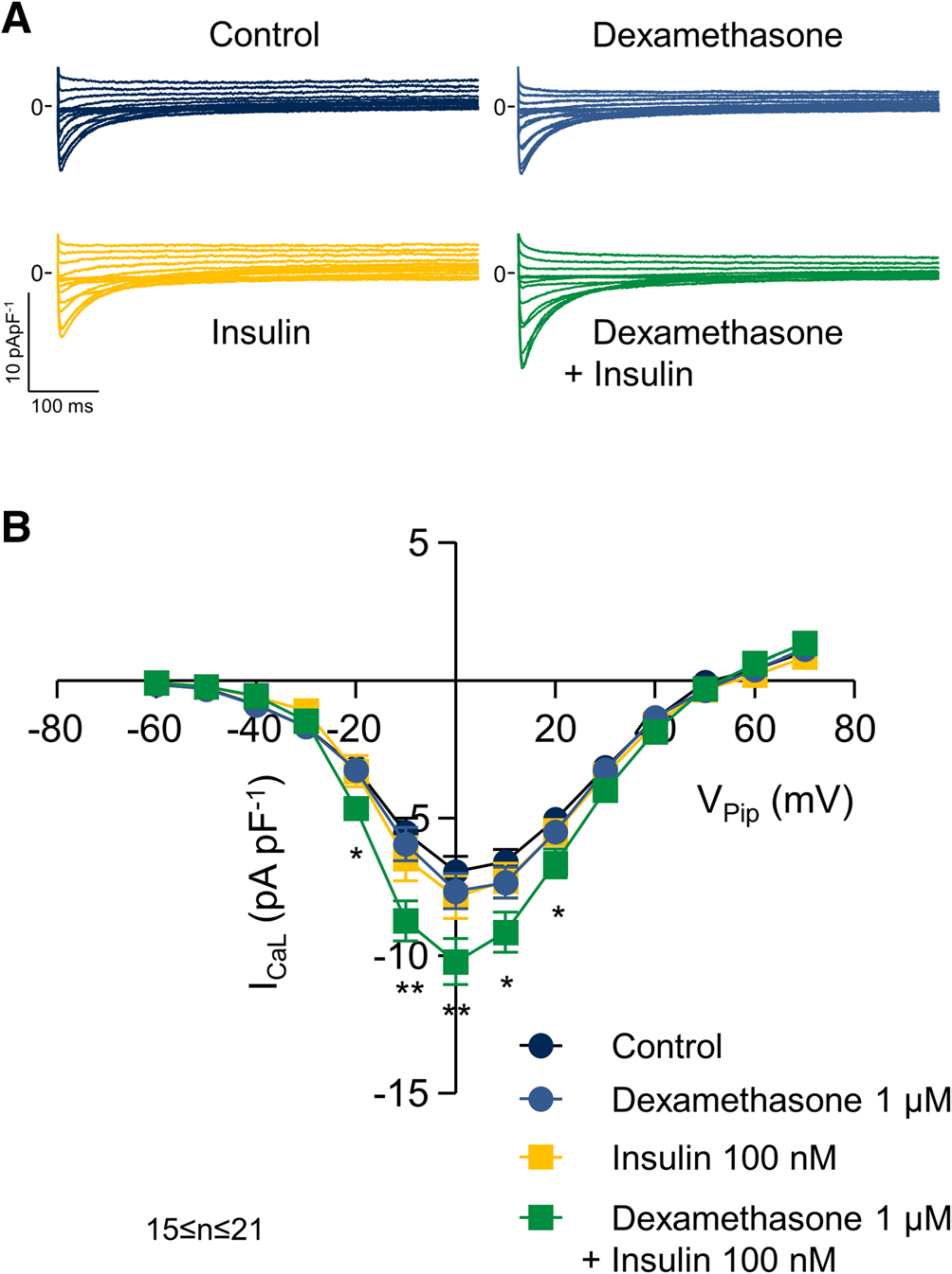
Dexamethasone increases I_CaL_ only in the presence of insulin. (**A**) Representative whole-cell current traces of I_CaL_ recorded from myocytes incubated for 24 h under control conditions (Control), with 1 µM dexamethasone, 100 nM insulin, 1 µM dexamethasone + 100 nM insulin. Myocytes were clamped for 600 ms from the holding potential of V_Pip_ = − 90 mV to test potentials between V_Pip_ = − 60 mV up to + 70 mV in steps of 10 mV. Na^+^ currents were inactivated by a prepulse of 70 ms to − 50 mV. Basic cycle length was 3,000 ms. (**B**) Average current–voltage relations of currents similar to those shown in A. I_CaL_ was quantified by subtracting the peak current from the current at the end of the voltage pulse (at 600 ms). *p < 0.05, **p < 0.01, dexamethasone + insulin versus control. 15 ≤ n ≤ 21.

**Figure 2:**
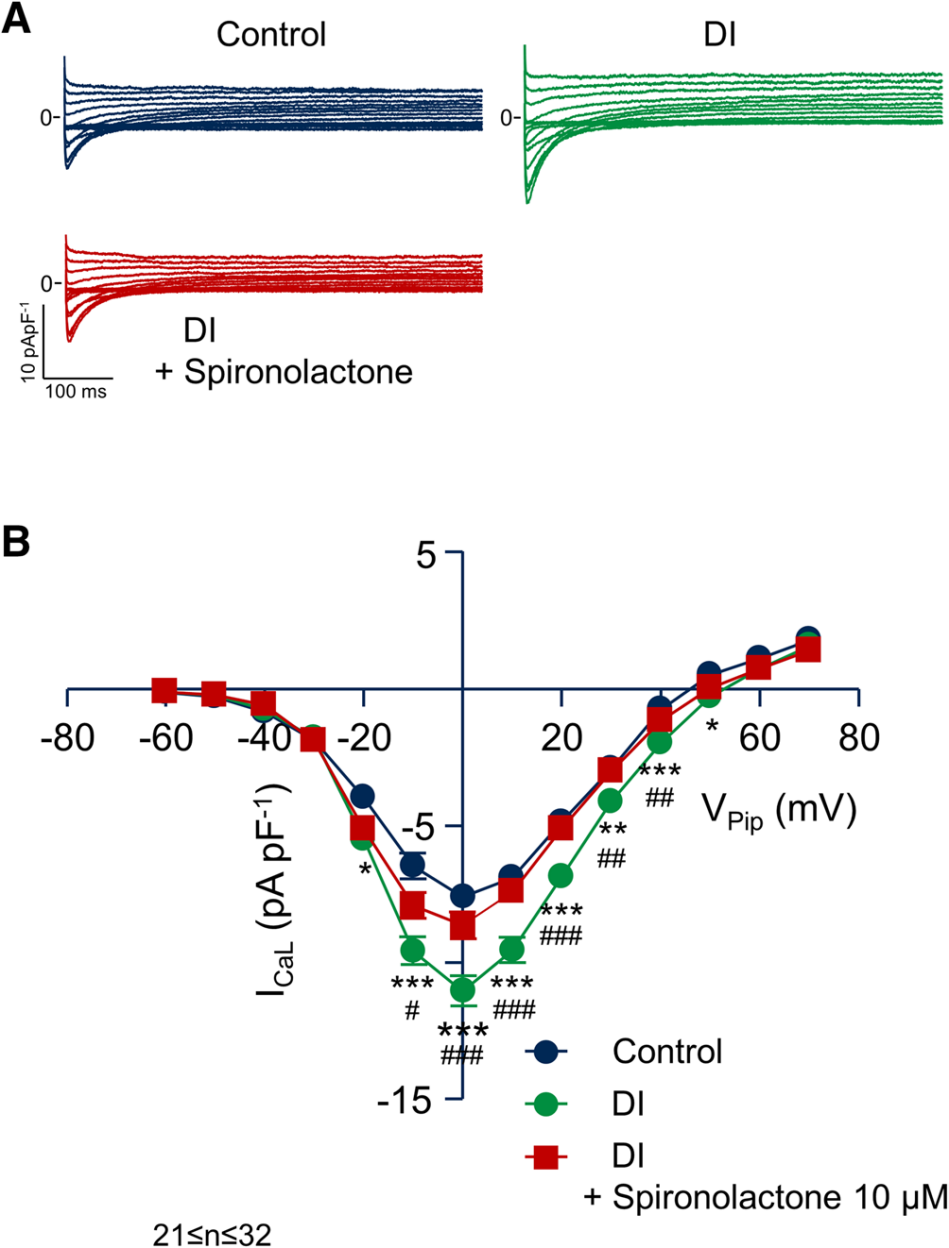
The effect of dexamethasone is mediated by the mineralocorticoid receptor. (**A**) Representative whole-cell current traces of I_CaL_ recorded from myocytes incubated for 24 h under control conditions (Control), with 1 µM dexamethasone + 100 nM insulin and 1 µM dexamethasone + 100 nM insulin + 10 µM spironolactone. Myocytes were clamped for 600 ms from the holding potential of V_Pip_ = − 90 mV to test potentials between V_Pip_ = − 60 mV up to + 70 mV in steps of 10 mV. Basic cycle length was 3,000 ms. (**B**) Average current–voltage relations of currents similar to those shown in (**A**). I_CaL_ was quantified by subtracting the peak current from the current at the end of the voltage pulse (at 600 ms). *p < 0.05, **p < 0.01, ***p < 0.001, dexamethasone + insulin versus control; ^#^*p* < 0.05, ^##^*p* < 0.01, ^###^*p* < 0.001 dexamethasone + insulin versus dexamethasone + insulin + spironolactone. 21 ≤ n ≤ 32.

Incubation of isolated left ventricular cardiomyocytes for 24 h with corticosteroids, such as the mineralocorticoid aldosterone or the glucocorticoid corticosterone, has been shown to increase the L-type Ca^2+^ current (I_CaL_) via activation of the mineralocorticoid receptor (MR)^8,27^. In the present study, we used dexamethasone as corticosteroid to stimulate I_CaL_. Moreover, in a previous study^27^, insulin was part of the primary culture conditions and we later learned that it is a prerequisite for I_CaL_ regulation by corticosteroids: Fig. 1A displays typical recordings of I_CaL_ obtained from a control myocyte, a myocyte incubated with 1 µM dexamethasone, a myocyte incubated with 100 nM insulin and a myocyte incubated with the combination of both dexamethasone and insulin. 24 h incubation with dexamethasone substantially increased I_CaL_ only in the presence of insulin. The current–voltage (I–V) relations shown in Fig. 1B summarize similar experiments. On average, dexamethasone + insulin increased I_CaL_ at 0 mV by 46% from 7.0 ± 0.5 pApF^−1^ (n = 21) to 10.2 ± 0.8 pApF^−1^ (n = 19, *p* < 0.01) while in the presence of dexamethasone (7.7 ± 0.6 pApF^−1^, n = 19) or insulin (7.9 ± 0.8 pApF^−1^, n = 15) alone, no significant difference was observed. For that reason we combined 1 µM dexamethasone with 100 nM insulin (DI) in all following experiments. To address the question whether DI increases I_CaL_ via the MR or the glucocorticoid receptor (GR), we incubated myocytes with DI alone and with DI and the MR antagonist spironolactone (10 µM). 24 h incubation with DI substantially increased I_CaL_, while in the presence of spironolactone, I_CaL_ remained at the level of the control myocytes (see Fig. 2A). The current–voltage (I–V) relations shown in Fig. 2B summarize similar experiments. Similar to the experiments shown in Fig. 1, DI increased I_CaL_ by 46% from 7.6 ± 0.4 pApF^−1^ (n = 21) to 11.1 ± 0.6 pApF^−1^ (V_Pip_ = 0 mV, n = 31, *p* < 0.001). In the presence of spironolactone, DI did not significantly affect I_CaL_, indicating that dexamethasone, like aldosterone or corticosterone^27^, increases I_CaL_ via the MR.

**Figure 3:**
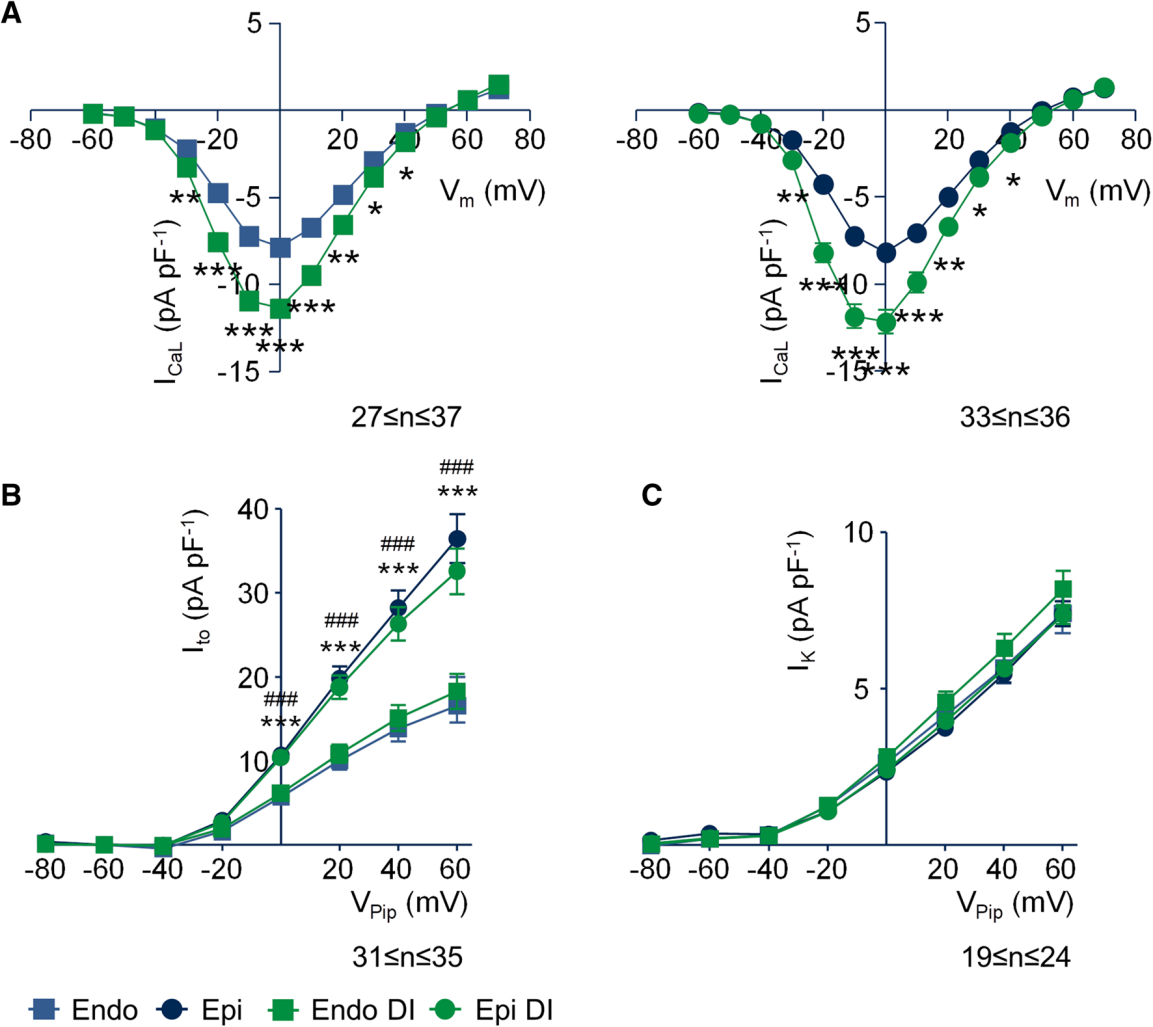
Effect of DI treatment on I_CaL_ and K^+^ currents in endo- and epicardial myocytes. (**A**) Average current–voltage relations of recordings similar to those shown in Fig. 1 and 2, obtained from endo- and epicardial myocytes of the left ventricular free wall incubated for 24 h under control conditions (blue) and with DI (green). *p < 0.05, **p < 0.01, ***p < 0.001, DI versus control. (**B** and** C**) Average current–voltage relations of I_to_ and I_K_ recorded from endo- and epicardial myocytes of the left ventricular free wall incubated for 24 h under control conditions (blue) and DI (green). Myocytes were clamped for 600 ms from the holding potential of V_Pip_ = − 90 mV to test potentials between V_Pip_ =  + 60 mV to − 80 mV in steps of −20 mV. Basic cycle length was 3,000 ms. I_to_ was quantified by subtracting the peak current from the current at the end of the voltage pulse (at 600 ms), I_K_ was estimated as the current at the end of the voltage pulse (600 ms). ***p < 0.001, epi- versus endocardial myocytes incubated under control conditions, ^###^*p* < 0.001, epi- versus endocardial myocytes incubated with DI.

To investigate regional differences in the effects of corticosteroids on I_CaL_, we isolated endo- and epicardial myocytes from the left ventricular free wall and investigated I_CaL_ after 24 h incubation with DI. Figure 3A shows average I–V relations of I_CaL_ recorded from endo (left panel) and epicardial (right panel) myocytes. Compared to control, DI increased I_CaL_ at 0 mV in endocardial and in epicardial myocytes to a similar extent by 45% (n = 37, *p* < 0.001) and 49% (n = 36, *p* < 0.001), respectively. We also investigated the effect of DI on repolarizing K^+^ currents. The transient outward K^+^ current (I_to_) displayed the typical gradient among the left ventricular free wall with much larger currents in epi-compared to endocardial myocytes. However, in both endo- and epicardial myocytes, I_to_ magnitude was not affected by 24 h incubation with DI (Fig. 3B). To address a potential effect of DI on the delayed rectifier group of K^+^ currents (I_K_), we used the current at the end of a 600 ms voltage pulse^15^. Figure 3C displays average I–V relations obtained by plotting the current at the end of a 600 ms voltage pulse versus the pulse potential. We did neither observe regional differences nor an effect of DI on I_K_.

**Figure 4:**
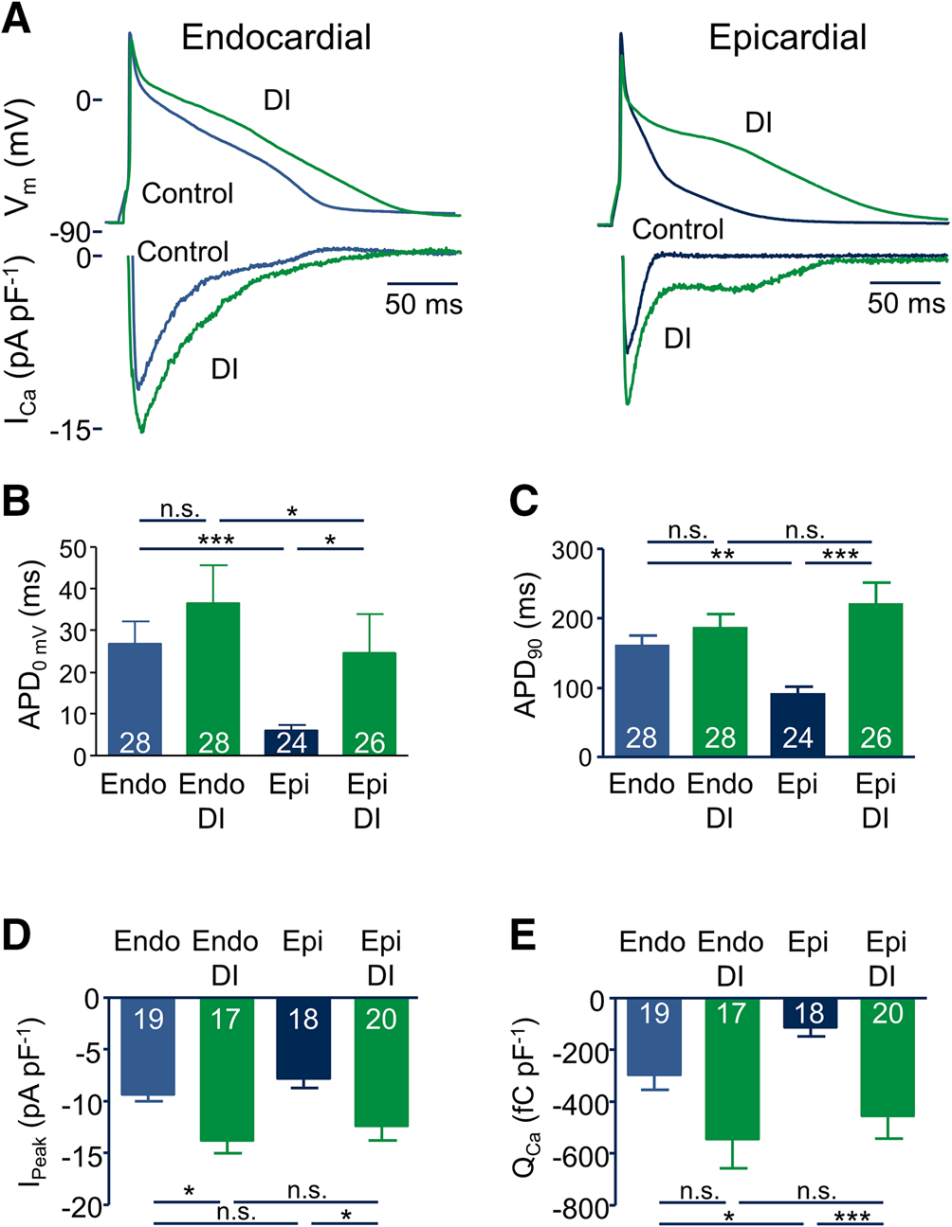
DI treatment abrogates the gradient in APD. (** A**) Representative APs and corresponding AP-induced Ca^2+^ currents recorded from endo- and epicardial myocytes incubated for 24 h under control conditions (blue) and with DI (green). APs were elicited by a train of suprathreshold depolarizing current injections at a basic cycle length of 1,000 ms. (**B**) Average APD at repolarization to 0 mV (APD_0mV_). (**C**) Average APD at 90% repolarization (APD_90_). (**D**) Average peak Ca^2+^ influx (I_Peak_) and (**E**) average total AP-induced Ca^2+^ influx (Q_Ca_) obtained from similar recordings to those shown in (** A**). Numbers in bars indicate number of myocytes in each group.

Next, we recorded action potentials (AP) from endo- and epicardial myocytes. Figure 4A (upper panels) shows representative AP recordings obtained under control conditions (blue) and after 24 h incubation with DI (green). As reported previously, APs were much shorter in epicardial than in endocardial myocytes. DI substantially increased APD in epicardial but not in endocardial myocytes. On average, in epicardial myocytes APD at 90% repolarization (APD_90_) increased from 90.2 ± 11.4 to 220.2 ± 31.4 ms (24 ≤ n ≤ 28, *p* < 0.001, Fig. 4C). In endocardial myocytes, the small increase in APD_90_ did not reach statistical significance. Similar results were observed with respect to the APD at 0 mV (Fig. 4B). The substantial increase in epicardial APD in the absence of alterations in endocardial APD completely abolished the large difference in APD between endo- and epicardial myocytes in the presence of DI.

Since under physiological conditions the ‘command potential’ for the I_CaL_ current is not a rectangular voltage pulse such the ones used in Figs. 1, 2 and 3, but rather the myocyte’s own AP, we conducted AP-clamp experiments to address the question whether the DI-induced changes in AP shape and duration affect the AP induced Ca^2+^ influx. We therefore recorded APs from myocytes and subsequently used their own individual AP as voltage template for the following voltage clamp experiments, in which we clamped the membrane voltage of each myocyte on its own AP in the absence and in the presence of 100 µM Cd^2+^ to inhibit I_CaL_. The resulting current is a good estimate of the AP-induced Ca^2+^ current^20^. Figure 4A (lower panels) displays the corresponding AP-induced Ca^2+^ currents obtained from individual myocytes of endo- and epicardial origin recorded under control conditions (blue) and after incubation with DI (green). Incubation with DI increased the peak AP-induced Ca^2+^ current in endo- and epicardial myocytes to a similar extent (Fig. 4D), reflecting the increase in I_CaL_ shown in Figs. 1, 2 and 3. The area under the AP-induced Ca^2+^ current equals the Ca^2+^ charge (Q_Ca_), i.e. the total amount of Ca^2+^, entering the myocyte via L-type channels during an AP. As we have published previously^20^, the long endocardial AP leads to a significantly larger Q_Ca_ in endo-compared to epicardial myocytes under control conditions. Incubation with DI dramatically increased Q_Ca_ by 301% (n = 18 vs. n = 20, *p* < 0.001) in epicardial myocytes, while in endocardial myocytes the increase was much smaller (84%, n = 19 vs. n = 20, n.s.) and did not reach statistical significance (Fig. 4C,E).

**Figure 5:**
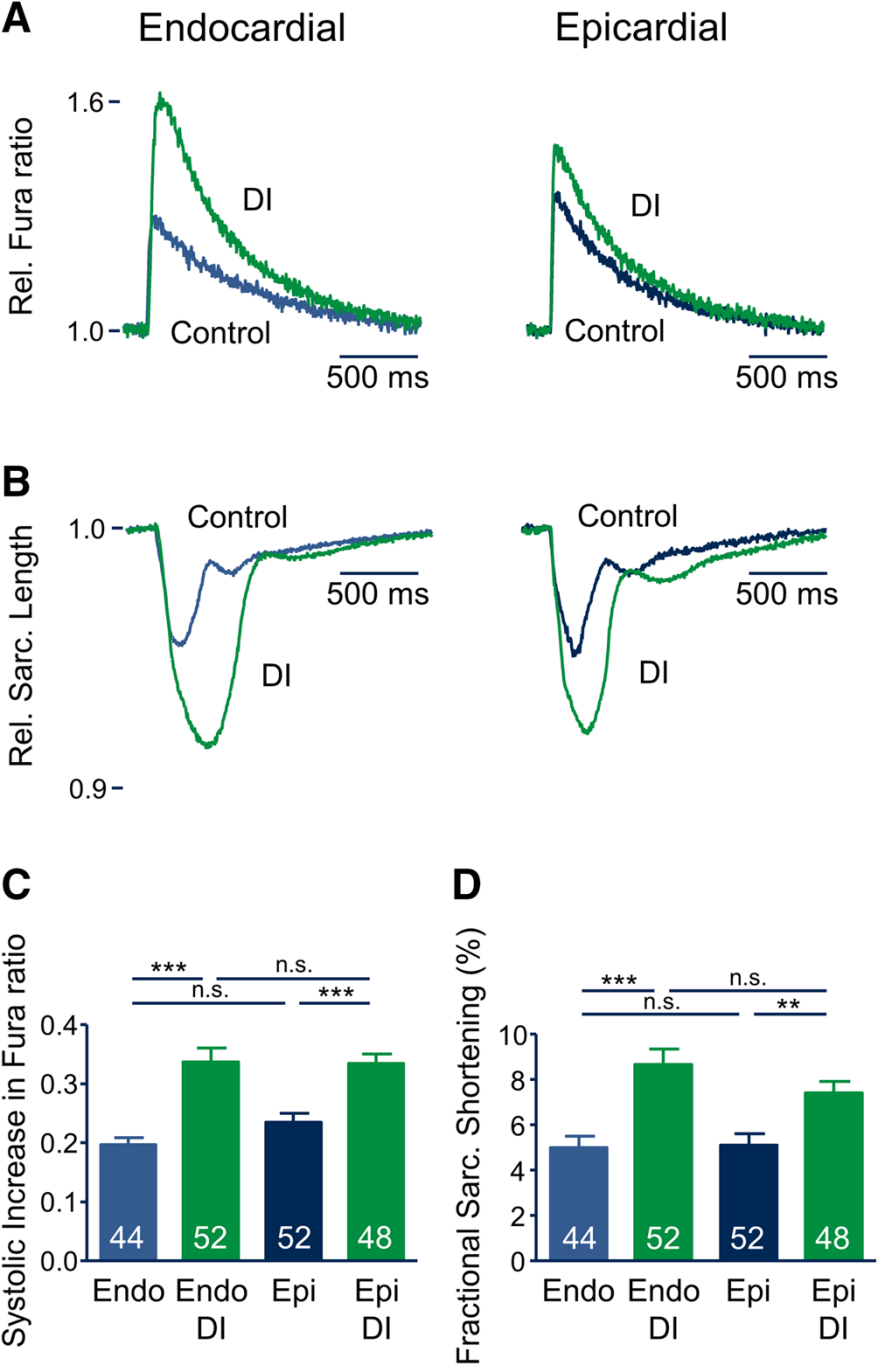
Effect of DI treatment on Ca^2+^ transients and contractility in endo- and epicardial myocytes. (**A**) Representative Ca^2+^ transient after 24 h incubation under control conditions (blue curve) and with DI (green curve). (**B**) Representative sarcomere length recordings of the same myocytes shown in** A. C**, average systolic increase in Fura ratio of recordings similar to those shown in (**A**), (**D**) average fractional sarcomere shortening of recordings similar to those shown in** A**. Numbers in bars indicate number of myocytes in each group.

**Figure 6:**
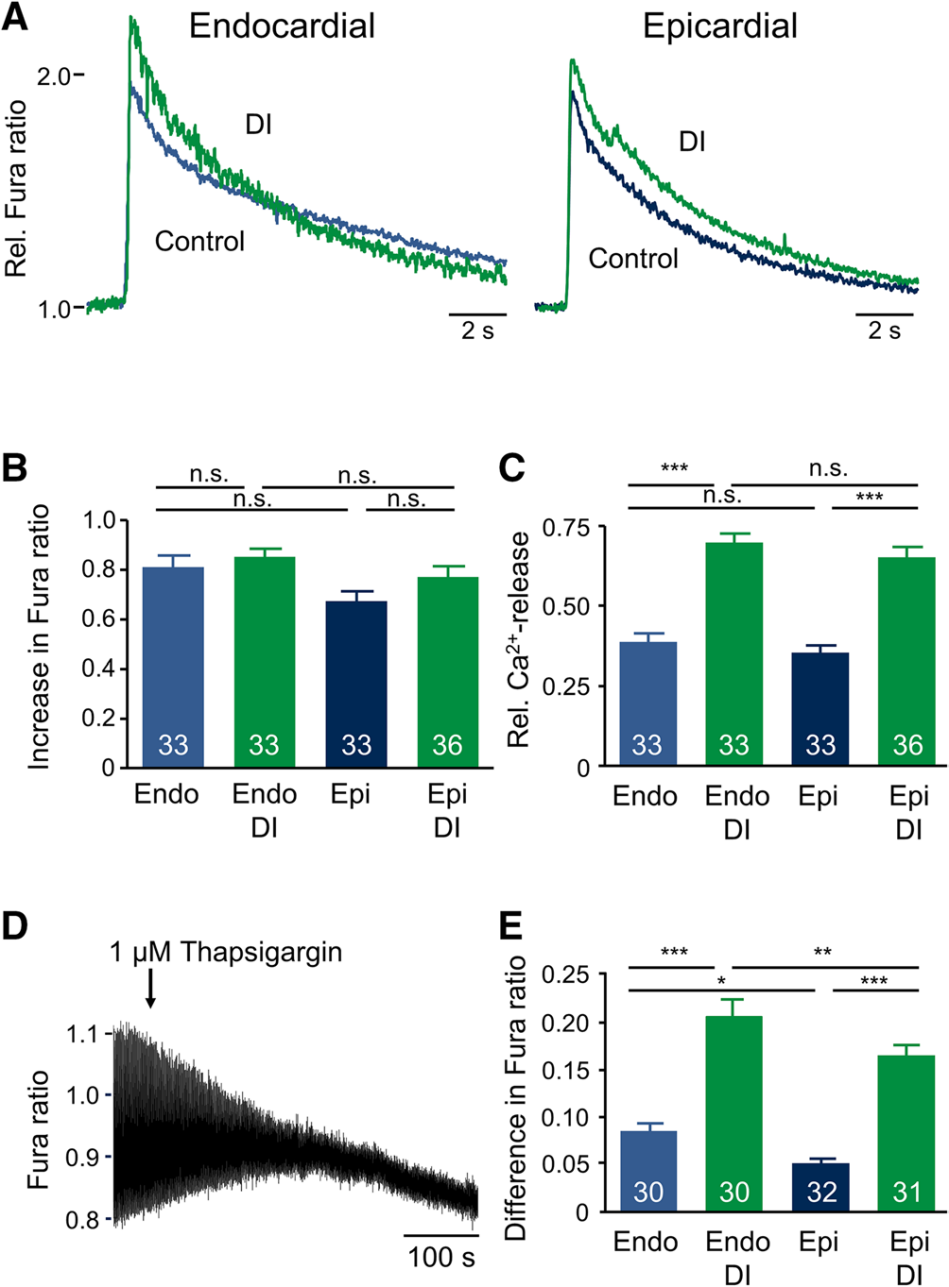
Effect of DI treatment on SR Ca^2+^ handling. (**A**) Representative caffeine-induced Ca^2+^ transients after 24 h incubation under control conditions (blue curves) and with DI (green curves). Caffeine (10 mM) was applied after 60 s of pacing at 1 Hz to ensure equal SR loading. (**B**) Average increase in Fura ratio in response to caffeine application. (**C**) Average relative Ca^2+^ release (Rel. Ca^2+^-release) calculated by dividing the increase in Fura ratio during regular pacing (1 Hz) by the increase in Fura ratio after application of caffeine. (**D**) Representative recording of Ca^2+^ transients after application of 1 µM thapsigargin obtained from a myocyte incubated under control conditions. (**E**) Average difference of systolic increase in Fura ratio calculated by subtraction of the systolic increase in Fura ratio after 5 min incubation with 1 µM thapsigargin from the systolic increase in Fura ratio before application of 1 µM thapsigargin. Numbers in bars indicate number of myocytes in each group.

Given the substantial increase in AP-induced Ca^2+^ influx, we combined Ca^2+^ imaging with simultaneous sarcomere length measurements to investigate intracellular Ca^2+^ transients and myocyte contractility. Figure 5A shows representative recordings of individual Ca^2+^ transients (given as relative Fura ratio) obtained from an endo- and an epicardial myocyte under control conditions (blue) and after 24 h incubation with DI (green), while Fig. 5B shows representative simultaneous recordings of sarcomere length. Despite the longer APD and the larger Q_Ca_ in endocardial cardiomyocytes, the amplitude of Ca^2+^ transients (endo: 0.20 ± 0.01, n = 48; epi: 0.23 ± 0.02, n = 52, n.s.) as well as fractional unloaded sarcomere shortening (endo: 5.0 ± 0.5%, n = 48; epi: 5.1 ± 0.5%, n = 52, n.s.) were similar in endocardial and epicardial myocytes after 24 h of incubation under control conditions (Fig. 6C+D). Similarly, diastolic Ca^2+^ level and diastolic sarcomere length were not significantly different between endo- and epicardial myocytes under control conditions (see Table 1). After 24 h incubation with DI, a substantial increase in the systolic Ca^2+^ transient was observed in endo- (+ 70%, 0.34 ± 0.02, n = 48, *p* < 0.001 vs. control) as well as in epicardial cardiomyocytes (+ 43%, 0.33 ± 0.02, n = 52, *p* < 0.001 vs. control). Accordingly, unloaded fractional shortening increased by 74% (8.7 ± 0.7%, n = 48, *p* < 0.001 vs. control) in endocardial cells and by 45% (7.4 ± 0.5%, n = 52, *p* < 0.05 vs. control) in epicardial myocytes (Fig. 5C and D). Diastolic Ca^2+^ levels and diastolic sarcomere length remained unaffected by DI (see Table 1).

**Table 1:** Effect of DI treatment on Ca^2+^ transients and contractility in endo- and epicardial myocytes.

	Endo	Epi	Endo DI	Epi DI
Diastolic Fura ratio	0.84 ± 0.01	0.82 ± 0.01	0.81 ± 0.01	0.84 ± 0.01
Systolic Fura ratio	1.14 ± 0.03	1.06 ± 0.03	1.40 ± 0.03***	1.35 ± 0.05***
Diastolic SL (µm)	1.56 ± 0.02	1.62 ± 0.02	1.53 ± 0.02	1.55 ± 0.02
Systolic SL (µm)	1.47 ± 0.02	1.57 ± 0.02	1.38 ± 0.02*	1.43 ± 0.02***

Diastolic and peak (Systolic) intracellular Ca^2+^ concentration (given as Fura ratio) and recorded from endo- and epicardial myocytes after 24 h incubation under control conditions or with DI. Diastolic and peak (Systolic) sarcomere length (SL) recorded during the same experiments. *p < 0.05, ***p < 0.001, DI versus control. 30 ≤ n ≤ 36.

To examine whether the increase in Ca^2+^ transient was due to an increased Ca^2+^ content of the SR, we analyzed Ca^2+^ transients evoked by rapid application of 10 mM caffeine after 60 s of steady-state pacing at 1 Hz. Figure 6A displays typical caffeine-induced Ca^2+^ transients recorded from endo- and epicardial myocytes under control conditions (blue) and after incubation with DI (green). Since caffeine locks the ryanodine-receptor in an open state, Ca^2+^ release from the SR is maximal^28^. Accordingly, the Ca^2+^ transients were substantially larger than those observed in response to pacing. SR Ca^2+^ content was assessed as the amplitude of the Ca^2+^ transients and was similar in endo- and epicardial myocytes under control conditions. Interestingly, DI did not significantly alter SR Ca^2+^ content (see Fig. 6B). However, in the presence of DI, the amplitude of the Ca^2+^ transients during regular pacing reached ~ 70% of the caffeine-induce Ca^2+^ transients, while under control conditions Ca^2+^ transients during pacing reached only ~ 30% of the caffeine-induced Ca^2+^ transients (Fig. 6C). This suggests that incubation with DI leads to a substantial increase in fractional release of Ca^2+^ from the SR.

To further address this question, we assessed Ca^2+^ transients under steady-state pacing at 1 Hz and then blocked Ca^2+^ uptake into the SR by inhibiting the SERCA using thapsigargin (1 µM) leaving residual Ca^2+^ transients arising from Ca^2+^ influx via L-type Ca^2+^ channels only. Figure 6D displays a typical recording of Ca^2+^ transients stimulated by 1 Hz pacing. The arrow indicates the application of thapsigargin to the bath solution. Within 3–5 min, Ca^2+^ transients had decreased to a residual amplitude reflecting Ca^2+^ influx from the extracellular space only. Figure 6E summarizes similar experiments and displays the difference between the amplitude of Ca^2+^ transients before and after the application of thapsigargin thus equaling the amount of Ca^2+^ released from the SR. In both, endo- and epicardial myocytes, DI substantially increased SR Ca^2+^ release. Since the total amount of Ca^2+^ in the SR was not affected (see Fig. 6B), this confirms that dexamethasone treatment increased the fractional release of Ca^2+^ from the SR during each cardiac cycle.

**Figure 7:**
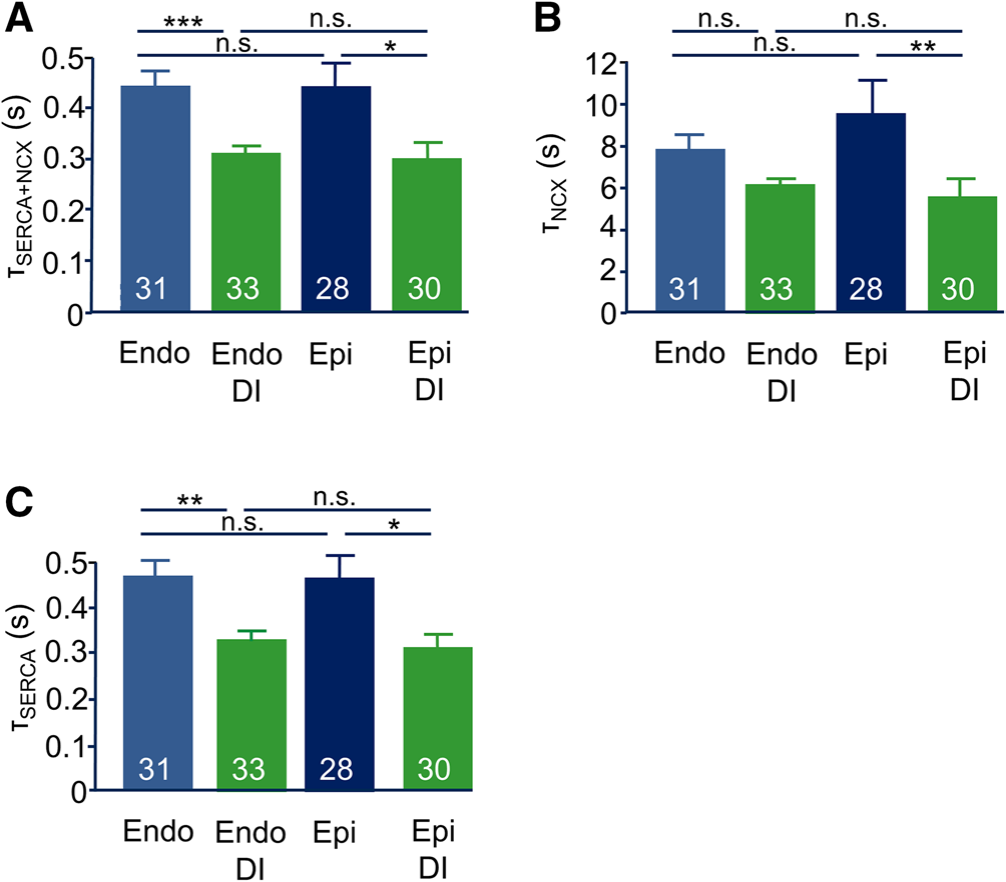
Effect of DI treatment on the time constants of cytosolic Ca^2+^ removal pathways. (**A**) Average Ca^2+^ transport time constants of SERCA + NCX (τ_SERCA + NCX_) estimated by monoexponential fitting of the decay of the Ca^2+^ transients recorded during regular pacing at 1 Hz. (**B**) Average Ca^2+^ transport time constants of NCX (τ_NCX_) obtained by monoexponential fitting of the decay of the Ca^2+^ transients in response to caffeine application. (**C**) Average average Ca^2+^ transport time constants of SERCA (τ_SERCA_) calculated by subtracting the NCX-rate constant calculated from caffeine-induced Ca^2+^ transients from the rate constants of the decay of Ca^2+^ transient during regular pacing. Numbers in bars indicate number of myocytes in each group.

In light of increased Ca^2+^ release from the SR and an increased Ca^2+^ influx from the extracellular space, both Ca^2+^ extrusion via the Na/Ca exchanger (NCX) and Ca^2+^ reuptake into the SR via SERCA should be increased in myocytes treated with DI. We therefore assessed time and rate constants of the decline of Ca^2+^ transients during regular pacing and in response to application of caffeine. In DI treated myocytes the time constant of the Ca^2+^ transient decay was accelerated in both endo- and epicardial myocytes (Fig. 7) indicating increased rates of Ca^2+^ removal from the cytoplasm. The time constant of the caffeine-induced Ca^2+^ transient decay was much slower, since in the presence of caffeine, Ca^2+^extrusion from the myocytes via the NCX is the only significant pathway left. In myocytes incubated with DI, the NCX time constants were accelerated compared to control which is consistent with the increased AP-induced Ca^2+^ influx upon DI treatment. The SERCA time constant (Fig. 7C, calculated as the reciprocal value of the difference in total and NCX-dependent rate-constants of the decay of the Ca^2+^ transient) was also substantially accelerated by DI treatment.

**Figure 8:**
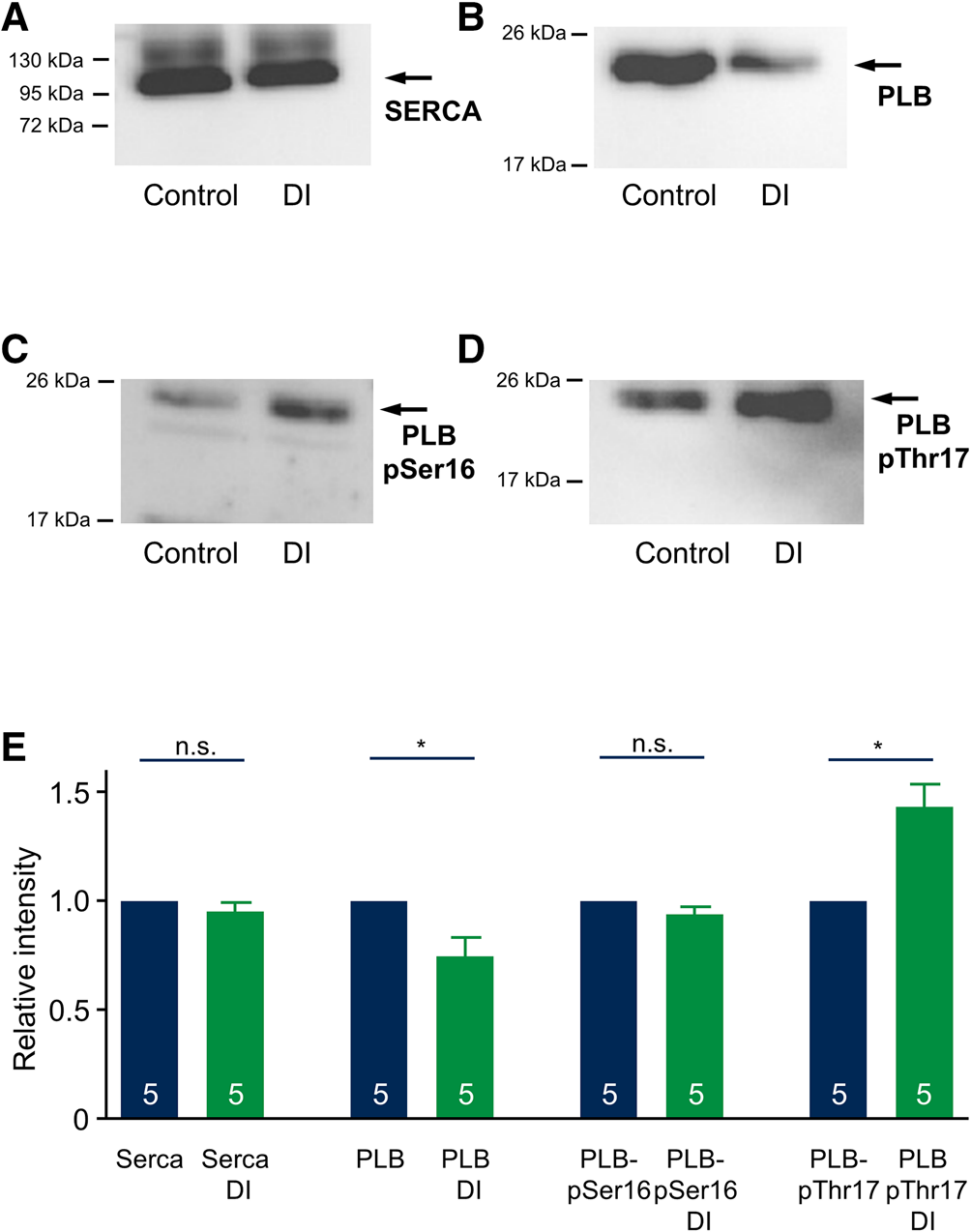
Effect of DI treatment on SERCA and PLB protein expression and phosphorylation. (**A-D**) Representative western blots stained for SERCA2 (**A**, 100 kDa), PLB (**B**, 25 kDa), phospho-PLB-Ser16 (**C**, 25 kDa) and phospho-PLB-Thr17 (**D**, 25 kDa). Protein was isolated from left ventricular myocytes incubated for 24 h under control conditions (Control) or with DI. Arrows indicate the according molecular weight level of the target protein. (**E**) Average relative protein expression was assessed by western blot quantification (normalization by Ponceau staining) of control (blue) and DI treated myocytes (green). n = 5 matched cell isolations. The full length gels of the western blots including the corresponding Ponceau stains are shown in the supplemental figure.

Finally, to address mechanisms underlying the increased SERCA transport rate, we performed western blot experiments in cardiomyocytes incubated for 24 h under control conditions and after treatment with DI to quantify SERCA and phospholamban expression as well as phospholamban phosphorylation. Since SERCA activity was similar in endo- and epicardial myocytes of both, control and DI groups, and in order to increase the total protein yield, we used isolated myocytes of the whole left ventricle for western blot experiments. Figure 8A–D display typical western blots stained against SERCA (8A), phospholamban (8B) and, using phospho-specific antibodies, against pSer16 (8C) and pThr17 (8D). Figure 8E summarizes the results of similar western blots and shows that SERCA expression is unaffected by treatment with dexamethasone while phospholamban expression was decreased. Moreover, phosphorylation of phospholamban increased at the Thr17 site thereby further reducing the inhibitory action of phospholamban on SERCA. This is consistent with increased SERCA activity.

## Discussion

In the present study, MR activation increased I_CaL_ to a similar extent in endo- and epicardial myocytes, demonstrating that, independent of regional origin, I_CaL_ is not only similar in magnitude, but is also identically affected by MR agonists. Although treatment with DI led to a much larger increase in APD and AP-induced Ca^2+^ influx in epicardial myocytes, the increase in intracellular Ca^2+^ transient and, hence, contractility was similar in both regions. Moreover, SR Ca^2+^ content was similar in both regions and not affected by DI treatment. We discovered that in both regions the fractional release of Ca^2+^ from the SR was increased by DI treatment. Our results suggest that the increase in I_CaL_ density rather than APD or AP-induced Ca^2+^ influx controls the increase in Ca^2+^ transient and contractility observed by DI treatment.

In the present study, we confirm regional differences in AP shape and duration as well as in underlying ionic currents that we and others have previously described for the left ventricular free wall of the rat^15,16,20^. Specifically, APD was substantially shorter in epicardial than in endocardial myocytes. This difference is attributable at least to a large extent to the larger epicardial I_to_ current density, while other ionic currents, such as I_K_ and I_CaL_, were similar in both regions. Since the AP waveform constitutes the voltage driving force for ionic currents including I_CaL_, the longer AP in endocardial myocytes led to an increased AP-induced Ca^2+^ influx in endocardial compared to epicardial myocytes, despite of a similar current density of I_CaL_ in both regions. This is in line with previous observations^20,29,30^ and underlines the importance of AP shape for control of the AP-induced Ca^2+^ influx. Moreover, all recordings in the present study were performed after at least 24 h incubation, hence, our data show that the regional differences in APD, underlying ionic currents and AP-induced Ca^2+^ influx remain well preserved even after a prolonged (24–36 h) period of primary culture.

Despite the larger AP-induced Ca^2+^ influx in endocardial myocytes, the intracellular Ca^2+^ transient as well as unloaded sarcomere shortening were similar in myocytes obtained from both regions. This fits well to previous observations in canine^31,32^ and rabbit^33^ left ventricle, while in mouse left ventricle a larger Ca^2+^ transient was found in endocardial myocytes^21,34^. For the rat left ventricle, conflicting results have been reported. Fowler et al. showed larger Ca^2+^ transients in endocardial myocytes probably evoked by an increased SR Ca^2+^ content^35^. However, they did not address myocyte contractility. On the other hand, Cazorla et al. reported similar Ca^2+^ transient amplitudes^36^ and Smail et al. also reported similar Ca^2+^ transient amplitudes and myocyte shortening in endo- and epicardial myocytes^37^. We also found no significant difference in Ca^2+^ transient amplitude or sarcomere shortening. Moreover, in our hands SR Ca^2+^ content was similar in endo- and epicardial myocytes, suggesting that the larger AP-induced Ca^2+^ influx in endocardial myocytes does not result in an increased SR Ca^2+^ filling state. In a state of intracellular Ca^2+^ homeostasis, a larger Ca^2+^ influx from the extracellular space into the cytoplasm during each AP must be matched by an increased Ca^2+^ extrusion into the extracellular space, carried predominantly by the NCX^38^. Consistently, we found a trend towards a shorter time constant of NCX Ca^2+^ removal in endocardial compared to epicardial myocytes (Fig. 7B), possibly indicating an increased NCX activity. This trend did not reach statistical significance, however, one should keep in mind that the difference in NCX time constant necessary to match the increased Ca^2+^ influx in endocardial myocytes without an increase in Ca^2+^ transient might be small and below our detection threshold, since the amount of Ca^2+^ entering via L-type current and exiting via the NCX is only a small fraction of the total Ca^2+^ transient. Moreover, by using EGTA in the pipette solution in our AP-clamp experiments we might have somewhat overestimated AP-induced Ca^2+^ influx since EGTA moderately ameliorates^39^ Ca^2+^-induced Ca^2+^-inactivation of I_CaL_.

It is well established that MR activation for > 18–24 h increases I_CaL_ in vitro in isolated cardiomyocytes^8,27^ as well as in vivo in mice with an increased plasma aldosterone concentration^40^ or in transgenic mice overexpressing the MR^11^. In the present study, we demonstrate that MR activation increases I_CaL_ in endo- and in epicardial myocytes to a similar extent. Accordingly, regulation of I_CaL_ magnitude by corticosteroids per se does not directly contribute to regional differences among the left ventricular free wall. However, especially in the early phase of the AP, the relation of the magnitudes of the repolarizing K^+^-current I_to_ and the depolarizing I_CaL_ not only sets the level of the plateau potential and controls early repolarization and the APD^26^ but is also a potent modulator of the Ca^2+^-influx during the AP. For example, an acute delay in early repolarization (e.g. caused by a decrease in I_to_) increased AP-induced Ca^2+^ influx^20,41^. One would therefore expect that an acute delay in early repolarization caused by an increase in I_CaL_ should even further increase the AP-induced Ca^2+^ influx, since not only the driving force (i.e. the membrane potential set by the AP) changes, but also the Ca^2+^ conductance of the membrane. Indeed, the increase in I_CaL_ we observed in response to MR activation substantially delayed early repolarization and increased APD, predominantly in epicardial myocytes, to such an extent that the endo-epicardial differences in APD disappeared. As a consequence, AP induced Ca^2+^-influx increased substantially in epicardial and to a lesser (but not significant) extent in endocardial myocytes. The relatively small increase of the AP induced Ca^2+^-influx in endocardial myocytes is consistent with their per se longer AP and delayed early repolarization, which leaves less room for an increase in APD upon an increase in I_CaL_.

MR activation led to a substantial increase in the intracellular Ca^2+^ transient in both endo- and epicardial myocytes. A prolonged AP with a resulting increase in sarcolemmal Ca^2+^ influx might alter several factors contributing to the intracellular Ca^2+^ transient: an increase in Ca^2+^ influx can trigger an increased release from the SR and might also increase the SR Ca^2+^ content which in turn increases SR Ca^2+^ release. Moreover, fractional release of Ca^2+^ from the SR is controlled by two mechanisms, the SR Ca^2+^ content and the magnitude of the trigger Ca^2+^, i.e. the AP-induced Ca^2+^ influx^38,42^. In the present study, we found no increase in SR Ca^2+^ content despite a substantial increase in AP-induced Ca^2+^ influx and in SERCA activity. The increase in amplitude of the Ca^2+^ transient can be explained by an increase in fractional release from the SR and (to a lesser extent) by an increase in trans-sarcolemmal Ca^2+^ influx. This finding is supported by previous observations. Trafford et al. showed that an increase in Ca^2+^ influx can substantially increase Ca^2+^ release from the SR (and contractility) in the absence of effects on SR Ca^2+^ content^43,44^. In animal models of heart failure with an increase in APD, Sah^41^ and Kaprielian^45^ observed an increased Ca^2+^ release from the SR in the absence of an increased SR Ca^2+^ content. In our study, fractional Ca^2+^ release averaged ~ 40% under control conditions which is similar to the ~ 50% observed by Picht et al*.*^46^. In cardiomyocytes from mice overexpressing the MR or after 48 h of incubation with aldosterone, SR Ca^2+^ content was also unaltered compared to control myocytes^7^. Since Bassani et al.^42^ found only a 4% increase in SR Ca^2+^ content in response to a switch to high loading condition, one could speculate that already under control conditions, SR Ca^2+^ content might be nearly maximal. Moreover, Bode et al.^47^ could show that in rat cardiomyocytes, SR Ca^2+^ content only weakly depends on SERCA activity, when the SR Ca^2+^ content is high. This was explained by an increase in SR Ca^2+^ leak. Interestingly, aldosterone has been shown to increase SR Ca^2+^ leak by downregulation of FKBP12 and 12.6 expression, thereby further limiting a potential increase in SR Ca^2+^ load^7^. SERCA activity is in addition modulated by SUMOylation^48,49^. SUMOylation of SERCA by SUMO1 is decreased in heart failure and contributes to decreased SERCA activity under this condition. Moreover, β-arrestin-2 enhances SERCA SUMOylation^49^. Interestingly, glucocorticoids decrease β-arrestin-2 expression at least in human lung adenocarcinoma cells^50^, could thus indirectly decrease SUMOylation of SERCA and thereby reduce SERCA activity. Taken together, modulation of I_CaL_ density via the MR appears as a potent regulator of intracellular Ca^2+^ transient magnitude and contractility in both, endo- and epicardial myocytes.

## Methods

### Isolation of myocytes

Cardiomyocytes were isolated from the left ventricular free wall of female Wistar rats (~ 220 g) as described previously^51^. After induction of deep anesthesia by intraperitoneal injection of thiopental-sodium (100 mg kg^−1^ body mass), the heart was quickly excised and placed into cold (4 °C) Tyrode’s solution. Subsequently, the aorta was retrogradely perfused for 5 min with modified Tyrode’s solution containing 4.5 mM Ca^2+^ and 5 mM EGTA (~ 1 μM free Ca^2+^ concentration) supplemented with 1 μM insulin (Sigma-Aldrich Chemie GmbH, Taufkirchen, Germany). The perfusion was continued for 19 min, recirculating 25 ml of the same solution containing collagenase (CLS type II, 160 U/ml, Biochrom KG, Berlin, Germany) and protease (type XIV, 0.6 U/ml, Sigma). Then, the heart was perfused for another 5 min with storage solution^8^ containing 100 μM Ca^2+^. Using fine forceps, tissue portions of the subendocardial (endocardial) and subepicardial (epicardial) layers were taken and placed in separate cell culture dishes containing the same solution at 37 °C. Tissue pieces were minced and gently agitated to obtain single cardiomyocytes. Myocytes were stepwise adapted to physiological Ca^2+^ levels, transferred to cell culture dishes containing storage solution supplemented with 1 g l^−1^ BSA, 100 IU ml^−1^ penicillin and 0.1 mg ml^−1^ streptomycin and stored at 37 °C in a water-saturated atmosphere containing 5% CO_2_. For incubation with dexamethasone and insulin, appropriate amounts of stock solutions containing dexamethasone (dissolved in 100% ethanol) and insulin were added to the respective cell culture dishes; the corresponding amount of ethanol was added to control groups, the final concentration of ethanol was ~ 0.02%. Isolated cardiomyocytes were used for experiments for up to 36 h. Only quiescent single rod-shaped cells with clear cross striations were used for experiments. All experiments were performed in accordance with relevant guidelines and regulations and all experimental protocols were approved by the *Regierung von Mittelfranken*, license No: 621–2,531.32–11/05.

### Patch-clamp technique

The ruptured-patch whole-cell configuration was used as described previously^15,52^. Myocardial cells were transferred into an elongated chamber (2.5 × 20 mm), mounted on the stage of an inverted microscope (Axiovert 25, Zeiss, Jena, Germany) and initially superfused with control solution. All experiments were performed at room temperature (22–25 °C). Patch pipettes were pulled from borosilicate glass (GC150-15, Clark Electromedical Instruments, Reading, UK) using a P-97 Puller (Sutter Instruments, Novato, CA, USA). Pipette resistance (R_Pip_) was 1.5—5 MΩ. Currents were recorded using an EPC-10 amplifier (HEKA Elektronik, Lambrecht, Germany), controlled by PULSE-Software (HEKA Elektronik). Membrane voltage (V_m_) and APs were recorded in the zero current-clamp mode and ionic currents in the voltage-clamp mode. For AP voltage-clamp recordings, APs were recorded at the beginning of the experiments and used as a voltage template in the voltage-clamp mode of the amplifier^20,53^. Membrane capacitance (C_m_) and series resistance (R_s_) were calculated using the automated capacitance compensation procedure of the EPC-10 amplifier. Series resistance was in the range of ~ 5 MΩ, was not allowed to exceed 10 MΩ and was compensated by 85%. The reference electrode of the amplifier headstage was bathed in pipette solution in a separate chamber and was connected to the bath solution via an agar–agar bridge filled with pipette solution. Pipette potential (V_Pip_) and V_m_ were corrected for liquid junction potentials at the bridge-bath junction. Whole-cell currents were low-pass filtered at 1 kHz and sampled at 5 kHz. Action potentials were sampled at 5 kHz.

To assess I_CaL_, myocytes were clamped for 600 ms from the holding potential of − 90 mV to test potentials between − 60 mV and + 70 mV in steps of 10 mV. Na^+^ currents were inactivated by a prepulse of 70 ms to − 50 mV. Basic cycle length was 3,000 ms. I_CaL_ was quantified by subtracting the current at the end of the test pulse from the peak current.^54^ To elicit outward K^+^ currents, myocytes were clamped for 600 ms from the holding potential of − 90 mV to test potentials between 60 mV and − 80 mV in steps of − 20 mV. Na^+^ currents were inactivated by a prepulse of 20 ms to − 50 mV. Basic cycle length was 3,000 ms. I_to_ was quantified by subtracting the current at the end of the test pulse from the peak current. I_K_ was defined as the current at the end of the voltage pulse.

### Ca^2+^ epifluorescence measurements

Ca^2+^ epifluorescence was recorded as previously described^55,56^. Cells were incubated in modified Tyrode’s solution with Fura-2-AM (4 μmol/l). Cells were transferred into a chamber (self-manufactured) mounted on an inverted microscope (Nikon DM IRB, Nikon, Düsseldorf, Germany). Ca^2+^ transients were recorded during field stimulation (1 Hz, 20–25 V, 4 ms duration; MyoPacer, IonOptix Corporation, Milton, MA, USA). Cells were alternatively excited at 340 and 380 nm (hyper-switch dual excitation, IonOptix Corporation). The F340/F380 ratio was used as an index of cytosolic Ca^2+^ concentration. In some experiments, caffeine or thapsigargin were washed in. For all Ca^2+^ fluorescence experiments, cells were paced for 1 min at 1 Hz before starting the measurements to ensure that Ca^2+^ balance was at steady state.

### Protein extraction

Isolated cardiomyocytes were incubated with DI for 24 h while myocytes isolated from the same heart were incubated for 24 h with vehicle and served as paired control. Myocytes were pelleted by centrifugation and dispensed in 1 ml TNE buffer. 40 µl protease inhibitor, 40 µl phosphatase inhibitor, 30 µl triton X-100 (10%), 5 µl PMSF (200 mM in 100% EtOH) and 20 µl sodium deoxycholate (12.5% in H_2_O) were added. Handled on ice at all times, samples were mechanically homogenized for 20 s, sonicated for 3 × 5 s and then centrifuged at 13,000 g and 4 °C for 10 min. The supernatant was used for further studies. To ensure equal protein loading, protein concentration was measured using the BCA Protein Assay Reagent Kit for microplate assay (Pierce, Rockford, USA). BSA in concentrations between 25 and 2000 µg/ml in TNE buffer was used as standard.

### Western blots

Samples were prepared for electrophoresis by adding Roti-Load 1 (4 × concentrated) (Carl Roth GmbH & Co. KG, Karlsruhe, Germany) at 1:4 to respective identical amounts of protein. Samples were heated to 95 °C for 5 min and then loaded pairwise (DI and the corresponding control) into the wells of 8% or 12% SDS polyacrylamide gels, separated by electrophoresis and blotted to PVDF membranes, which were blocked in TBST solution containing 1% dry milk and probed for SERCA and phospholamban. Primary antibodies were: goat SERCA2 (C-20): sc-8094 (Santa Cruz Biotechnology, Santa Cruz, USA), mouse phospholamban: A010-14 (Badrilla Ltd., Leeds, United Kingdom), rabbit phospholamban pSer16: A010-12 (Badrilla Ltd.), rabbit phospholamban pThr17: A010-13 (Badrilla Ltd.). Secondary antibodies were: goat anti-rabbit (1:50,000, polyclonal, Santa Cruz), rabbit anti-goat (1:50,000, polyclonal, Sigma), goat anti-mouse (1:50,000, polyclonal, Santa Cruz). Blots were developed using the Super Signal West Femto Maximum Sensitivity Substrate (Thermo Fisher Scientific, Waltham, MA, USA). Chemiluminescence was acquired using a STELLA imager with the XStella and AIDA image analyzer software (raytest Isotopenmeßgeräte, Straubenhardt, Germany). Image saturation was prevented by adjusting exposure times of the camera. Using serial dilutions we ensured for each antibody that the applied amounts of protein were within the linear detection range of the imaging system. The amount of total protein was assessed using Ponceau S staining^57^.

### Solutions

For the isolation of myocytes, modified Tyrode’s solution contained (in mM): NaCl 138, KCl 4, Glucose 10, NaH_2_PO_4_ 0.33, MgCl_2_ 1, HEPES 10, CaCl_2_ 4.5, EGTA 5, titrated to pH 7.30 using NaOH. The same solution without EGTA and with 2 mM Ca^2+^ was used as bath solution for the patch-clamp and fluorescence imaging experiments. For cell digestion, collagenase (162.8 U/ml, CLSII, Biochrom AG, Berlin, Deutschland) and protease (0.54 U/ml, type XIV, Sigma-Aldrich GmbH, Steinheim, Deutschland) were added to modified Tyrode’s solution (10^–6^ M Ca^2+^). Storage solution contained (in mM): NaCl 130, NaH_2_PO_4_ 0.4, NaHCO_3_ 5.8, MgCl_2_ 0.5, CaCl_2_ 1, KCl 5.4, glucose 22, and HEPES 25, titrated to pH 7.40 with NaOH in the presence of 5% CO_2_ and supplemented with 1 mg ml^−1^ BSA. For recording action potentials and K^+^-currents, the pipette solution contained (in mM): glutamic acid 120, KCl 10, MgCl_2_ 4, EGTA 10, HEPES 10, and Na_2_ATP 2, pH 7.20 with KOH. For recording Ca^2+^ currents, the pipette solution contained (in mM): CsCl 130, MgCl_2_ 5, EGTA 10, HEPES 10, Na_2_ATP 2, pH 7.20 with CsOH.

For protein extraction and western blots, TNE buffer contained (in mM): Tris 20, NaCl 150, EDTA 1, pH = 7.40 and was completed with protease inhibitor (Complete Mini, Roche, 1 tablet for 10 ml buffer) and PMSF (1 mM) just before starting protein extraction. SDS sample buffer was the mix from the homogenizing buffer and 4xRoti-Load (Roth, Karlsruhe, Germany). TBST solution contained (in mM): Tris 50, NaCl 150, 0.05% Tween-20, pH 7.6 with HCl.

### Data analysis and statistics

Patch clamp data were analyzed using the PULSE-FIT software (HEKA Elektronik, Lambrecht/Pfalz, Germany), IGOR Pro (WaveMetrics, Lake Oswego, USA), and Microsoft Excel (Microsoft Corporation, Redmond, USA) as described previously^54^. Ca^2+^ epifluorescence data were analyzed using Ionwizard 5.0 (IonOptix Corporation, Milton, USA) and Microsoft Excel. Quantitative densitometric analysis of western blots was performed using ImageJ software. The intensity of specific bands was determined after background subtraction and normalized to the total protein content per lane as quantified by densitometric analysis of the corresponding Ponceau stains^58^.

Data are given as mean ± SEM. Statistical significance was evaluated by paired or unpaired Student's *t* test when two groups were compared or one-way ANOVA followed by Newman-Keuls test (with the exception of APD_0mV_ and Q_Ca_, which were analyzed by Kruskal Wallis test followed by Dunn's post test because both were not normally distributed) when more than two groups were compared using Prism 5 (GraphPad, San Diego, USA). *p* < 0.05 was considered statistically significant.

## Supplementary information

Supplementary information 1
