# Supplementary material for: Region-specific mechanisms of corticosteroid-mediated inotropy in rat cardiomyocytes
Source: Sci Rep. 2020 Jul 14;10:11604. doi: 10.1038/s41598-020-68308-4 (PMC7360564; doi:10.1038/s41598-020-68308-4)
Supplement: Supplementary file 1 — Supplementary information 1 [file 41598_2020_68308_MOESM1_ESM.pdf]

Region-Specific Mechanisms of Corticosteroid-Mediated Inotropy in Rat Cardiomyocytes

by

Caroline Wacker, Niklas Dams, Alexander Schauer, Anne Ritzer, Tilmann Volk and Michael Wagner

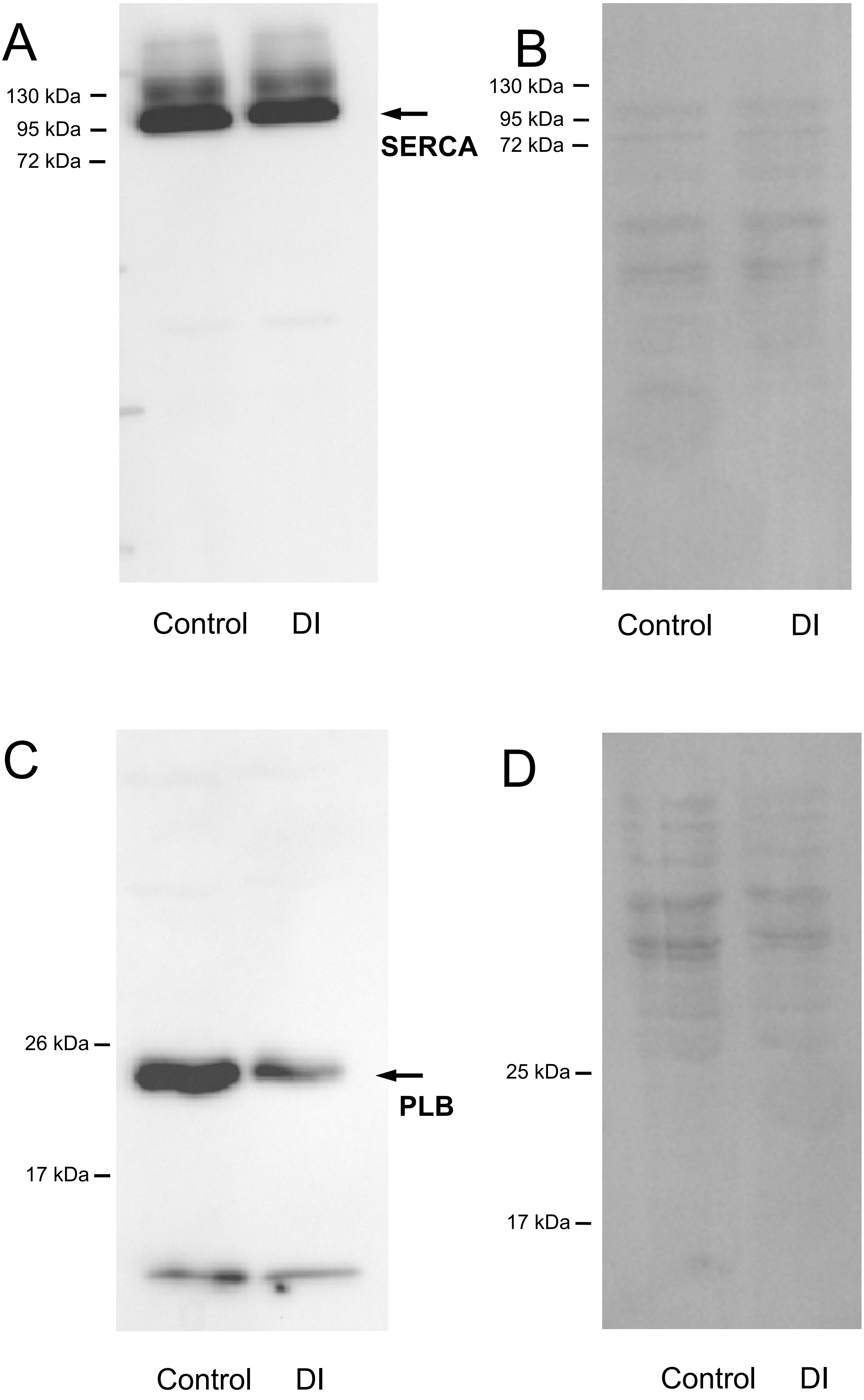

Supplemental Fig. 1

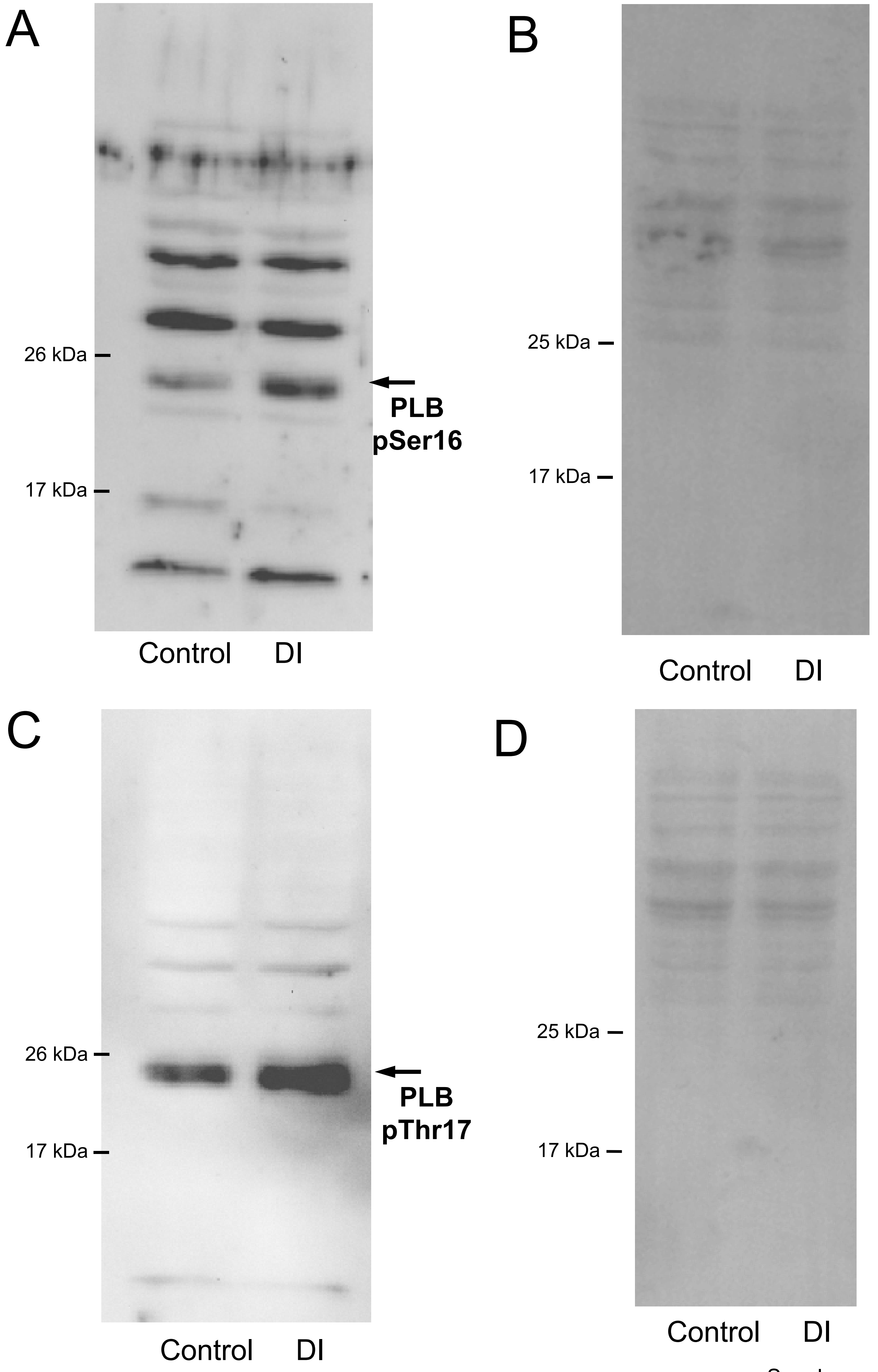

Supplemental Fig. 2

Supplemental Figure 1: Effect of DI treatment on SERCA and PLB protein. A+B, representative western blot stained for SERCA2 (A, 100 kDa) and corresponding Ponceau stain of the same blot (B). C+D, representative western blot stained for PLB (C, 25 kDa) and corresponding Ponceau stain of the same blot (D). Protein was isolated from left ventricular myocytes incubated for 24h under control conditions (Control) or with DI. Arrows indicate the according molecular weight level of the target protein.

Supplemental Figure 2: Effect of DI treatment on PLB phosphorylation. A+B, representative western blot stained for phospho-PLB-Ser16 (A, 25 kDa) and corresponding Ponceau stain of the same blot (B). C+D, representative western blot stained for phospho-PLB-Thr17 (C, 25 kDa) and corresponding Ponceau stain of the same blot (D). Protein was isolated from left ventricular myocytes incubated for 24h under control conditions (Control) or with DI. Arrows indicate the according molecular weight level of the target protein.
